# Expression of CXCL10 is associated with response to radiotherapy and overall survival in squamous cell carcinoma of the tongue

**DOI:** 10.1007/s13277-013-1549-6

**Published:** 2014-01-07

**Authors:** Matilda Rentoft, Philip John Coates, Lotta Loljung, Torben Wilms, Göran Laurell, Karin Nylander

**Affiliations:** 1 Department of Chemistry, Umeå University, 901 85 Umeå, Sweden; 2 Tayside Tissue Bank, Medical Research Institute, Ninewells Hospital and Medical School, University of Dundee, Dundee, UK; 3 Department of Medical Biosciences/Pathology, Umeå University, Building 6M, 2nd Floor, 901 85 Umeå, Sweden; 4 Department of Clinical Sciences/Otorhinolaryngology, Umeå University Hospital, 901 87 Umeå, Sweden; 5 Department of Surgical Science/ENT, Uppsala University Hospital, 75185 Uppsala, Sweden

**Keywords:** Oral cancer, Tongue, Chemokines, CXCL10, Radiotherapy, Prognosis

## Abstract

Five-year survival for patients with oral cancer has been disappointingly stable during the last decades, creating a demand for new biomarkers and treatment targets. Lately, much focus has been set on immunomodulation as a possible treatment or an adjuvant increasing sensitivity to conventional treatments. The objective of this study was to evaluate the prognostic importance of response to radiotherapy in tongue carcinoma patients as well as the expression of the CXC-chemokines in correlation to radiation response in the same group of tumours. Thirty-eight patients with tongue carcinoma that had received radiotherapy followed by surgery were included. The prognostic impact of pathological response to radiotherapy, N-status, T-stage, age and gender was evaluated using Cox’s regression models, Kaplan-Meier survival curves and chi-square test. The expression of 23 CXC-chemokine ligands and their receptors were evaluated in all patients using microarray and qPCR and correlated with response to treatment using logistic regression. Pathological response to radiotherapy was independently associated to overall survival with a 2-year survival probability of 81 % for patients showing a complete pathological response, while patients with a non-complete response only had a probability of 42 % to survive for 2 years (*p* = 0.016). The expression of one CXC-chemokine, CXCL10, was significantly associated with response to radiotherapy and the group of patients with the highest CXCL10 expression responded, especially poorly (*p* = 0.01). CXCL10 is a potential marker for response to radiotherapy and overall survival in patients with squamous cell carcinoma of the tongue.

## Introduction

Squamous cell carcinoma (SCC) in the oral cavity is one of the ten most common malignancies worldwide, with the tongue being the most tumour prone sub-site. The relatively low 5-year survival, of around 50 %, has remained disappointingly stable over the last few decades in spite of improvements in the main treatments of surgery and radiotherapy [1–3]. This has evoked a rising interest in identifying biomarkers that are able to predict prognosis and response to treatment [2]. Today the most informative marker is node status, N-status, where cervical lymph node involvement drastically worsens the prognosis [4, 5]. A complicating factor is the fairly high rate of occult cervical nodal metastasis, which is especially frequent in tumours of the tongue (∼30 %) [3].

Several factors are known to influence response to radiotherapy in head and neck cancer patients, including tumour characteristics (e.g. location, volume and tumour stage), patient characteristics (e.g. smoking status) and biological factors (e.g. hypoxia and expression of DNA repair genes) [6–9]. The potential of the natural immune response for improving response to conventional treatments has recently been in focus [10–12]. The goal is to enhance the anti-tumoural specific response, which can be achieved either by nonspecific or specific stimulation of the immune system. The immune response towards tumours is, however, a complex process, involving both pro- and anti-tumour components and research on the interaction between tumours and the microenvironment is still fairly young.

Chemokines are small secreted immune modulators representing a large family of proteins that were initially characterized as attractants of leucocytes. They signal through G-protein-coupled (chemokine) receptors and are divided into four subgroups (CC, CXC, CX3C and C), depending on structure, with CC and CXC representing the major classes. CXC-chemokines were initially closely related to angiogenesis and are further divided into angiogenic and angiostatic but are also reported to be important for cell survival and metastasis [12]. Several CXC-chemokine ligands activate more than one receptor, and the majority of the receptors bind several CXC-ligands. They are induced by inflammatory cytokines, growth factors and pathogenic stimuli and are produced and secreted by many different cell types including tumour cells and tumour-infiltrating immune cells.

In addition to their importance for tumour survival, metastasis and angiogenesis, chemokines have recently been implicated in treatment response [12]. A number of studies have shown a considerable effect of radiation on chemokine expression, and there are indications for a role for chemokines in resistance to chemo- and radiotherapy [13–16]. Chemokines are interesting molecular targets due to their properties as natural immune modulators, and monoclonal antibodies against chemokine receptors have been used in experimental settings to inhibit growth and spread of malignant tumours [17, 18]. The role and expression of many chemokines are not yet well established in oral cancer, but a study mapping the expression of 24 chemokine ligands and receptors in a number of SCC head and neck cell lines before and after radiation was recently published. This study showed large variation in chemokine receptor and ligand expression, and further evaluation of chemokines as biomarkers for radiation response was suggested to be of value [19].

The aim of this study was to summarize the expression of CXC-chemokines in tongue tumours and investigate the relationship to radiation response. Additionally, the association between radiation response and 5 year survival in tongue tumour patients was assessed, as response to preoperative treatment has prognostic value in some other solid tumours, including tonsillar carcinoma [20–22]. Results showed a strong association between radiation response and overall survival for tongue tumour patients and identified CXCL10 as a candidate chemokine for predicting radiation response.

## Patients and methods

### Patients

Thirty-eight patients for which response to radiotherapy could be evaluated were identified from a previous study on stromal inflammation and tongue cancer [23]. These patients had received and completed preoperative radiotherapy, and surgery and pathological evaluation of the response to radiotherapy were performed on surgical specimens at the ENT Clinic, Norrlands University Hospital. The pathological response was judged as complete (cPR) if no viable cancer cells could be detected and as incomplete (non-cPR) if viable cancer cells were detected. Patients were classified as young if diagnosed before 40 years of age and classified as old if over 40 years at diagnosis, in accordance with the literature [24–29]. The study was approved by the local ethics committee (dnr 01-210; dnr 08-003M)

### Array data

Microarray data from samples taken at diagnosis for all 38 patients had previously been made publically available at gene expression omnibus (GEO) (accession number GSE34115), and data for 16 of the 17 CXC-chemokine ligands and all 7 chemokine receptors were extracted [30]. Even though data for 24 more tongue tumours were available, we focused on the 38 patients for which pathological response to radiotherapy could be evaluated. The chemokine ligand CXCL15 was not available on the microarray chip and therefore not included in the study. Array data for normal tongue tissue from 16 controls also included in the above study was used for comparison.

### qPCR data

Expression of the chemokine ligand CXCL10 was evaluated using qPCR in all samples. Relative quantities were calculated using the ΔCT method. The gene TUBA6, previously demonstrated to be stably expressed in oral FFPE tissue, was used as reference gene [31, 32]. cDNA reactions were performed using RevertAid H minus first strand cDNA kit (Fermentas Gmbh, Leon-Rot, Germany) with 200 ng of RNA. For the PCR reactions, the quanti tect primer assay was used for CXCL10 and in-house designed primers for TUBA6 together with the quanti tect SYBR green assay (Qiagen Gmbh, Hamburg, Germany) [32].

### Statistical analysis

Correlations between the 2-year survival and age, gender, T-stage, N-status and pathological response to radiotherapy were investigated by chi-square tests, and Cox’s regression models were used to assess their prognostic value. Survival curves were drawn using the Kaplan-Meier method, and the log rank test was used to assess the survival difference between groups.

Fold changes and significances (tumour vs control) for the CXC-chemokines were calculated using array data. A value above or below the mean expression of controls plus/minus two standard deviations (mean control ±2*SD) was used as a cut-off to decide how many tumours had increased/decreased expression of the separate CXC-chemokines. Array data for all CXC-chemokines and qPCR data for CXCL10 were used to correlate expression levels to pathological response to radiotherapy using logistic regression, both in a univariate analysis and a multivariate model correcting for gender, age, size of tumour and nodal status. Expression of CXCL10 was additionally categorized into three similar sized groups, low (*n* = 13), medium (*n* = 12) and high expression (*n* = 13), to further evaluate its relationship to pathological response to radiotherapy. The significance level was set to *p* < 0.05, and all analysis were performed using SPSS software version 19.0 (SPSS Inc., Chicago, IL, USA)

## Results

### Clinical data

Clinical data is summarized in Table 1. The male to female ratio was 1.2:1, and most patients presented with a T1 or T2 tumour (76 %). The age span was 19–81 years, with a mean age of 50 years, which is slightly lower than the typical patient group. This could partially be caused by a larger proportion of the older patients being judged as unfit for surgery in contrast to more of the younger patients completing both radiotherapy and surgery. Pathological response to radiotherapy was evaluated in surgical samples from all 38 patients; 26 patients showed complete response (cPR) and 12 did not (non-cPR). For three patients with non-cPR, the tumour was not radically removed at surgery. One of these patients went through extended surgery, showing tumour-free resection margins, and two were treated with cytostatics. All patients had been followed for at least 2 years or until death.

**Table 1:** Summary of patients data

	Number (%)
Age at diagnosis	
Mean	50
Range	19–81
Gender	
Male	21 (55)
Female	17 (45)
Tumour size	
T1	10 (26 )
T2	19 (50 )
T3	7 (19)
T4	2 (5)
Nodal status^a^	
N−	29 (78 )
N+	8 (22 )
Pathological response to radiotherapy	
cPR	26 (68 )
Non-cPR	12 (32 )
2-year survival	
Yes	26 (68)
No	12 (32 )
5-year survival	
Yes	18 (47)
No	15 (40 )
NA^b^	5 (13 )

^a^One patient had unknown N-status

^b^Five year follow-up not yet passed

### Importance of complete response to preoperative radiotherapy for overall survival

**Fig. 1:**
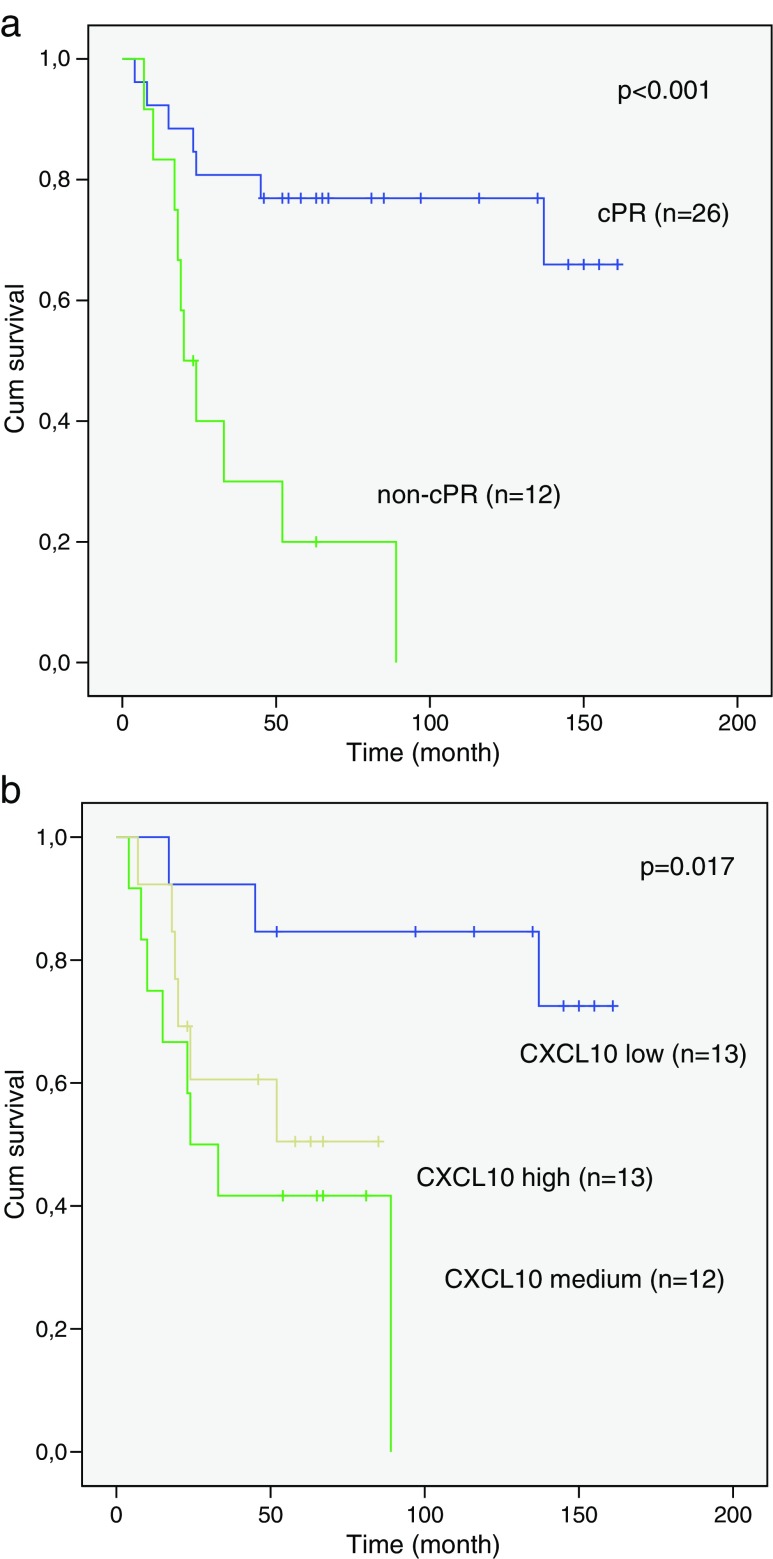
Kaplan-Meier survival curve showing **a** the relationship between pathological response to radiotherapy (cPR vs non-cPR) and overall survival. **b** The relationship between grouped CXCL10 expression (low, medium, high) and overall survival censoring of patients is shown as *vertical lines*

Univariate survival analysis by Cox’s regression of the variables pathological response to radiotherapy, gender, age, T-stage or N-status identified pathological response to treatment as the only variable significantly correlating to overall survival (hazard ratio (HR) 95 % confidence interval (CI), 6.0 (2.1–16.9), *p* = 0.001). Multivariate analysis further demonstrate that pathological response to radiotherapy was an independently unfavourable prognostic factor (HR 95 % CI, 5.3 (1.7–16.9), *p* = 0.005). Chi-square test showed that a cPR was already important for the 2-year survival (*p* = 0.016), with 81 % probability of surviving 2 years when having a complete response to radiotherapy as compared to 42 % for patients with non-cPR. Survival curves for patients with a non-cPR as compared to patients with cPR can be found in Fig. 1a and it illustrates a significantly poorer overall survival for patients with a non-cPR (*p* < 0.001, log rank test). After removing the three patients for whom initial surgery did not radically remove the tumour from the analysis, results remain significant (*p* = 0.002, log rank test).

### Expression of the CXC family of chemokines and their receptors in tongue tumours

**Fig 2:**
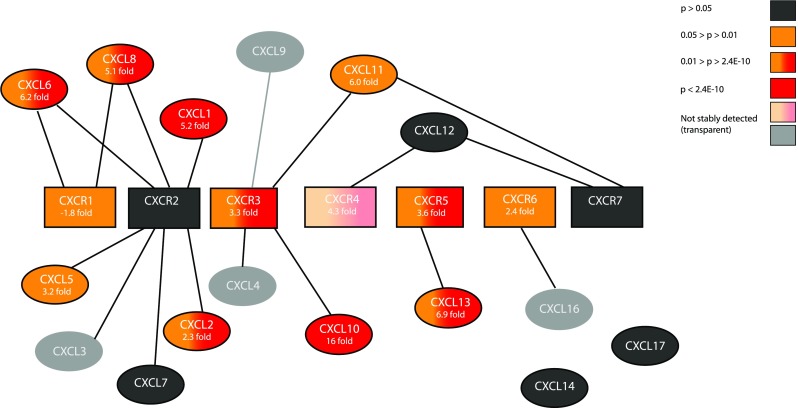
Summary of microarray expression data for all CXC-chemokine ligands (*oval*) and receptors (*squares*), except CXCL15, in tongue tumours compared to normal controls. *Colour* indicates significance as defined in the figure. *P* < 2.4E-6 is the Bonferroni corrected significance level recommended when all genes in a 20 818 gene array is tested. Only two genes fall below this level. Fold changes can be found in each shape

RNA from all 38 patients and probes for detection of all but one of the 24 CXC-chemokine ligands and receptors were included in a previously published microarray study made public on GEO [30], and their expression in tongue tumours as compared to controls is summarized in Fig. 2. Five of the CXC-chemokines (CXCL3, 4, 9 and 16 and CXCR4) had a detection *p* value above 0.05 (caused by low signal or low stringency), and data for these should thus be interpreted with care. Eight CXC-chemokine ligands were significantly up-regulated (CXCL1, 2, 5, 6, 8, 10, 11, 13), and none were significantly down-regulated. Of the receptors, three were significantly up-regulated (CXCR3, 5, 6) and one (CXCR1) significantly down-regulated. CXCL10 had the highest fold change with an average increase of 16-fold. For some chemokines, a changed expression (>mean control ±2*SD) could be seen in almost all patients, while other chemokines were only increased or decreased in a subgroup of patients summarized in Table 2.

**Table 2:** Percentage of samples with increased/decreased expression of the significant CXC-ligands and receptors

Gene	%
CXCR6	43
CXCR1^a^	50
CXCL5	57
CXCL2	61
CXCL8	68
CXCR5	68
CXCR3	71
CXCL11	71
CXCL1	82
CXCL10	89
CXCL13	89
CXCL6	93

^a^The only gene showing decreased expression in tumours

### Association between the expression of CXCL10 and response to radiotherapy

Using continuous array data and logistic regression to investigate the association between CXC-chemokine ligand and receptor expression in response to radiotherapy, one chemokine, CXCL10, was found to be significant (*p* = 0.03), with higher levels resulting in a poorer response to radiotherapy.

Because of the somewhat noisy nature of microarray data, CXCL10 levels were analysed using qPCR to obtain more accurate expression values before further evaluating the relationship with radiotherapy response. The correlation between the two methods (qPCR and microarray) was good (*R* = 0.76). In a multivariate logistic regression model, correcting for gender, age, size of tumour and nodal-status, CXCL10 expression was again significantly associated with response to radiation therapy (*p* = 0.05). Categorising samples into three equal groups according to CXCL10 expression (low, medium, high) showed that patients with the highest expression of CXCL10 had significantly poorer pathological response to radiotherapy compared to patients expressing the lowest levels (*p* = 0.01) (Table 3). Survival analysis of patients according to the categorised CXCL10 expressions showed significantly better survival in the group with the lowest expression of CXCL10 (*p* = 0.017, log rank test) (Fig. 1b).

**Table 3:** Logistic regression after categorising CXCL10 expression into low, medium and high

	*p* value	OR and 95 % CI	
CXCL10 expression (high vs low)	0.01	19.2	(1.88–196.53)
CXCL10 expression (high vs medium)	0.07	4.8	(0.86–26.79)

## Discussion

In spite of the easily accessible location of tumours in the oral cavity, both from a diagnostic and a therapeutic point of view, survival is fairly low and patients die from metastatic disease, loco-regional recurrence and second primary tumours. In this study, we found that complete response to preoperative radiation is important for survival in tongue cancer patients and that CXCL10 expression could indicate resistance to radiotherapy.

Response to preoperative treatment has previously been shown to be a strong prognostic marker for a number of cancers. In a large study on 167 tonsillar carcinomas, Friesland and co-workers found that the probability of surviving 5 years when displaying complete response to radiotherapy was 79 %, while it was only 9 % for patients showing incomplete response (*p* = <0.0001) [21]. Similar results were achieved when looking at cervical cancer stage Ib and IIa and preoperative radiotherapy (5-year survival cPR = 95 % and non-cPR = 46 %, *p* < 0.0001) as well as breast cancer and preoperative chemotherapy (5-year survival cPR = 88 % and non-cPR = 77 %, *p* < 0.033) [20, 22]. In our cohort of tongue tumour patients, a cPR after preoperative radiotherapy resulted in a 2-year survival of 81 %, while patients with a non-cPR only had 42 % probability of surviving 2 years (*p* = 0.016), confirming the importance of radiotherapy response also in these patients.

N-status is a well-known predictor of prognosis, and the fact that no significance was obtained in this study is probably partially due to the small number of N+ patients (*n* = 8). The high number of expected occult metastasis in tongue tumours (∼30 %) also decreases the sensitivity of the analysis [3]. The survival of young tongue cancer patients is a much debated subject, and there are as many studies showing no difference or an even better prognosis in young patients as there are studies showing a poor prognosis [24–29, 33]. In this small cohort of 38 patients, we did not see any significant difference in overall survival between young and old patients.

CXC-chemokines has previously been connected to response to treatment, but their roles in cancerogenesis differ between tumour types. We therefore wanted to clarify which CXC-chemokines have a changed expression in tongue tumours and their relation to radiation resistance. The tongue additionally differs morphologically from other tissues in the oral cavity, and it has recently been shown that both normal and malignant tongue tissue are molecularly distinct from other tissues within the region, indicating that collectively analysing all tissues in the oral cavity as done historically could be misleading [34, 35]. In our study, eight CXC-chemokine ligands and three receptors were significantly up-regulated and one receptor was significantly down-regulated in tongue tumours. Expression of one of the CXC-ligands, CXCL10, was strongly associated to radiation response. Categorising CXCL10 expression into three groups showed that it was especially patients with the highest expression of CXCL10 that respond poorly to radiotherapy.

CXCL10 has an unclear role in cancer. It is angiostatic and as expected has anti-tumour characteristics [36]. Intratumoral injection of CXCL10 leads to reduced growth and impaired angiogenesis and metastasis in murine adenocarcinoma and studies have shown a synergistic effect against tumours through its immunomodulatory properties in murine models of glioma and melanoma [37–39]. Both CXCL10 and its receptor CXCR3 are on the other hand over-expressed in many tumours and have been connected to poor prognosis and metastasis in a number of cancers, including colon cancer, multiple myeloma, breast cancer and basal cell carcinoma [40–44]. Simultaneous expressions of CXCL10 and CXCR3 in breast cancer cell lines additionally lead to CXCL10-dependent proliferation of CXCR3-positive cells and treatments using CXCR3 antagonists, and ligand-neutralizing antibodies inhibit metastasis in melanoma and breast cancer in mice [45–47]. Explanations to the dual role of CXCL10 as both a tumour inhibitor and a tumour promoter can be many; the receptor CXCR3, for example, exists in three isoforms (CXCR3-A CXCR3-B and CXCR3-alt) with different outcomes upon activation, and the balance between the three could be of importance; structural properties of CXCL10 are thought to be significant for in vivo activity, and the effect of CXCL10 signalling is suggested to vary depending on whether the microenvironment or the tumour cells express the receptor [36, 48, 49]. Treatments targeting CXCL10 therefore have to be used with care today, and it is important to fully understand the role of CXCL10 in individual tumour types and subgroups of tumours.

CXCR1 is the only chemokine in our study showing significant down-regulation. CXCR1 together with CXCR2 represent the two angiogenic receptors even though CXCR2 is considered the major angiogenic receptor in humans [50]. The two ligands for CXCR1, CXCL6 and CXCL8, which also activate CXCR2, are both up-regulated, indicating that a shift towards activation of CXCR2 could be of importance for tongue carcinoma. Antibody-based inhibition of CXCR2 in pancreatic cancer models has previously been shown to block angiogenesis with an accompanying reduction in tumour growth, and a recent study of CXCR2 and oral cancer showed antagonist inhibition of CXCR2 to decrease tumour cell viability [51–53].

The main limitation of this study is the restricted number of available samples. We included all tongue tumour patients treated at Norrlands University Hospital between 1998 and 2010 that had gone through preoperative radiotherapy and surgery and for which there were an available tumour sample from diagnosis. Still, only 12 patients with a non-cPR were available and confirmation of our result in an independent dataset will be important. The arrays from which we retrieve the expression data contains probes for 20 818 genes. If we were to test them all, the very stringent and commonly used Bonferroni correction would be applied, and the significance level would be set to *p* < 2.4E-6. This would result in only CXCL1 and CXCL10 being considered significantly up-regulated (Fig. 2). The Bonferroni correction ensures that the majority of the identified genes in large-scale experiments are true positives but simultaneously reduces the sensitivity of the analysis. The present study was, even though utilizing microarray data, based on a prior hypothesis and investigating a limited number of genes, and we therefore did not take multiple testing into consideration.

In conclusion, we have comprehensively characterized the expression of the CXC-chemokine ligands and their receptors in tongue tumours. We have correlated their expression to pathological response to radiotherapy and found that especially a group of patients with highly increased expression of CXCL10 is associated with non-complete response, indicating a role for this gene in resistance to radiotherapy. Additionally, we have confirmed the importance of cPR for overall survival of tongue carcinoma patients. Taken together, our data identify high-level CXCL10 expression as a predictive biomarker for poor response to radiotherapy in tongue cancers. These patients may therefore benefit from alternative treatments, possibly involving specific targeting of CXCL10.
